# The contribution of two isozymes to the pyruvate kinase activity of *Vibrio cholerae*: One K^+^-dependent constitutively active and another K^+^-independent with essential allosteric activation

**DOI:** 10.1371/journal.pone.0178673

**Published:** 2017-07-07

**Authors:** Carlos Guerrero-Mendiola, José J. García-Trejo, Rusely Encalada, Emma Saavedra, Leticia Ramírez-Silva

**Affiliations:** 1 Departamento de Bioquímica, Facultad de Medicina, Universidad Nacional Autónoma de México, Ciudad de México, México; 2 Departamento de Biología, Facultad de Química, Universidad Nacional Autónoma de México, Ciudad de México, México; 3 Departamento de Bioquímica, Instituto Nacional de Cardiología, Ignacio Chávez, Ciudad de México, México; Cinvestav-IPN, MEXICO

## Abstract

In a previous phylogenetic study of the family of pyruvate kinase EC (2.7.1.40), a cluster with Glu117 and another with Lys117 were found (numbered according to the rabbit muscle enzyme). The sequences with Glu117 have been found to be K^+^-dependent, whereas those with Lys117 were K^+^-independent. Interestingly, only γ-proteobacteria exhibit sequences in both branches of the tree. In this context, it was explored whether these phylogenetically distinct pyruvate kinases were both expressed and contribute to the pyruvate kinase activity in *Vibrio cholerae*. The main findings of this work showed that the isozyme with Glu117 is an active K^+^-dependent enzyme. At the same substrate concentration, its *V*_max_ in the absence of fructose 1,6 bisphosphate was 80% of that with its effector. This result is in accordance with the non-essential activation described by allosteric ligands for most pyruvate kinases. In contrast, the pyruvate kinase with Lys117 was a K^+^-independent enzyme displaying an allosteric activation by ribose 5-phosphate. At the same substrate concentration, its activity without the effector was 0.5% of the one obtained in the presence of ribose 5-phosphate, indicating that this sugar monophosphate is a strong activator of this enzyme. This absolute allosteric dependence is a novel feature of pyruvate kinase activity. Interestingly, in the K^+^-independent enzyme, Mn^2+^ may “mimic” the allosteric effect of Rib 5-P. Despite their different allosteric behavior, both isozymes display a rapid equilibrium random order kinetic mechanism. The intracellular concentrations of fructose 1,6-bisphosphate and ribose 5-phosphate in *Vibrio cholerae* have been experimentally verified to be sufficient to induce maximal activation of both enzymes. In addition, Western blot analysis indicated that both enzymes were co-expressed. Therefore, it is concluded that *Vc*IPK and *Vc*IIPK contribute to the activity of pyruvate kinase in this γ-proteobacterium.

## Introduction

*Vibrio cholerae* is a prokaryotic bacterium that contains two unique circular chromosomes [1]. It has three copies of genes (*pyk*F, *pyk*A-1and *pyk*A-2) encoding three distinct pyruvate kinases (PKs) EC (2.7.1.40), respectively here referred to *Vc*IPK, *VcIIPK* and *Vc*IIIPK. Two of these genes (*pyk*F and *pyk*A-1) are present in chromosome 1 (genome size 2.96 Mb) and the other gene (*pyk*A-2) is located in chromosome 2 (1.07 Mb) [2]. A previous phylogenetic analysis of the PK family showed a dichotomic tree structure separating the family of the PKs into two clusters [3]. Cluster I groups 52.6% of the total sequences analyzed, and is characterized by having a glutamate at position 117 (numbered according to the rabbit muscle enzyme (RMPK)) and cluster II with 46% of total sequences having a lysine at the same position. To date, all characterized PKs harboring Glu117 exhibit K^+^-dependent activity, whereas those with Lys117 are K^+^-independent. *Vc*IPK is located in cluster I, and *Vc*IIPK in cluster II, suggesting that they should exhibit K^+^-dependent and K^+^-independent activity, respectively. The feature of having isozymes in both branches of the phylogenetic tree is thought to be unique to γ-proteobacteria. On the other hand, most PKs are regulated by heterotropic effectors whose nature depends on the cell metabolism [4, 5]. Cluster I is mainly comprised of PKs modulated by bi-phosphorylated sugars such as fructose 1,6 bisphosphate (Fru 1,6-BP) [6, 7] or fructose 2,6 bisphosphate (Fru 2,6-BP) [8,9] whereas cluster II includes enzymes largely regulated either by AMP or monophosphate sugars [10–12]. Members of the γ-proteobacteria group such as *Escherichia coli* [10], *Salmonella typhimurium* [13] and *Yersinia enterocolitica* [14] also have both type I and type II PKs which are located, respectively in Cluster I and Cluster II of the phylogenetic tree. These enterobacteriaceae type I PKs are modulated by Fru 1,6-BP, and the type II are regulated principally by AMP. In this vein, we were interested in elucidating whether *Vc*IPK and *Vc*IIPK, which are phylogenetically distant, were expressed, and contributed to the pyruvate kinase activity of *Vibrio cholerae*. Since there are no previous studies on PKs from this γ-proteobacterium, we determined their kinetic and regulatory properties and explored their expression in *V*. *cholerae*. The data show, that *Vc*IPK is a constitutively active enzyme, whereas *Vc*IIPK is mediated by an allosteric activation. However, since its allosteric effector was present in *V*. *cholerae* and both enzymes were co-expressed, it was concluded that both *Vc*IPK and *Vc*IIPK contribute to the activity of pyruvate kinase in this γ-proteobacterium.

## Materials and methods

### Cloning, expression and purification of *Vc*IPK and *Vc*IIPK

The PK genes from *Vibrio cholerae* chromosome 1 were amplified from genomic DNA extracted from the *V*. *cholerae* N16961 strain. The genes were amplified by PCR using the following primers *for Vc*IPK: FW 5’-TACTTCCAATCCAATGCTAAAAAGACCAAAATCGTATGTACGATT-3’ and RV 5’-TTATCCACTTCCAATGTTACAGTAGGTGTACTGATGCAGTGTT-3’ and for *Vc*IIPK: FW 5’-TACTTCCAATCCAATGCTAGCTCAACTTTGCGTAGAACAAAAAT-3’ and RV 5’-TTATCCACTTCCAATGTTAATAGACAGGTAAGATGCGCATACA-3’. Each insert was introduced into the pMCSG7 vector as described in [15]. The plasmids were isolated and sequenced to verify the absence of mutations. The resulting pMCSG7/*Vc*IPK or pMCSG7/*Vc*IIPK plasmids were used to transform *E*. *coli* BL21-CodonPlus (DE3)-RIL competent cells. Both proteins were then overexpressed in four liters of Luria Bertani (LB) medium then growth, induced, harvested and lysed as described previously [15]. The suspensions were centrifuged at 13,250 × *g*, the supernatants loaded on a His Trap FF column (GE Healthcare), and the enzymes eluted with a linear gradient of imidazole (10–500 mM). The fractions with the highest activity of PK were pooled (200 mM imidazole), concentrated by membrane filtration (Centricon 100,000 molecular weight cutoff), and loaded on a Hi Trap desalting column (GE Healthcare). Both enzymes were precipitated with ammonium sulfate at 80% saturation and stored at 4°C. Unfortunately, despite several attempts, the poly-histidine tag was not able to be removed using TEV protease for either enzyme, indicating that the cleavage site is not accessible to the TEV protease. Therefore, in subsequent purifications the incubation with TEV protease was omitted for both enzymes. The *Vc*IPK and *Vc*IIPK were 90 and 88% percent pure, respectively, as determined by SDS-PAGE (Laemmli [16] at 12.5% polyacrylamide; 4°C and 160 V) by densitometry analysis after application of Coomassie brilliant blue stain. The yield for both enzymes was ~40 mg per 4 liters of cell culture. Both proteins were positively identified by mass spectrometry using a BRUKER microflex MALDI-TOF mass spectrometer (spectra not shown) to obtain the grade of purity and molecular mass of the isozymes. To determine the enzyme oligomeric state, 50 μg of *Vc*IPK and *Vc*IIPK were loaded onto a blue native-PAGE (BN-PAGE) and separation was carried out overnight at 4°C as described in [17] with a gradient gel of 4–11% acrylamide, 100 μg of solubilized extract of lauryl maltoside inverted membranes of *Paracoccus denitrificans* and 100 μg of mitochondrial extract of rat liver were used as molecular weight markers.

To probe if the His_6_ tag effects the kinetic behavior of *V*. *cholerae* PKs. *Vc*IPK was sub-cloned into pTrc99A and transformed into *E*. *coli* PB25Δ-PKIΔ-PKII. Cells were lysed by sonication. The crude extract was precipitated with 50 and 90% ammonium sulfate. After overnight dialysis, the sample was loaded in a size-exclusion column (Sephacryl 300) equilibrated with 25 mM HEPES containing 50 mM KCl (pH 7.0). Fractions with the highest activity were collected and loaded to a cation-exchange column (Carboximethyl-Sepharose) in 25 mM HEPES pH 7.0. The protein eluted in the front, and fractions with the highest activity were then loaded into an anion-exchange column (Diethylaminoethyl-Sepharose), and both PKs eluted with a linear gradient of 25 mM HEPES containing 1M KCl (pH 7.0). SDS-PAGE consistently showed that PK co-purified with other proteins. Assays of activity of this preparation revealed that the kinetic constants for PEP^3-^ and ADP-Mg of *Vc*IPK with and without the His_6_tag were almost identical. To further compare enzymatic activity, the same ionic strength was used (150 mM). *K*_0.5_ for PEP^3-^ was 0.55 ± 0.015 mM and 0.68 ± 0.016 mM for the His_6-_tagged and non His_6-_tagged protein, respectively. *K*_0.5_ for ADP-Mg was 0.26 ± 0.03 mM and 0.27 ± 0.02 mM for the His_6-_tagged and non His_6-_tagged protein, respectively. The response to the allosteric effector was similar. In the presence of 2 mM Fru 1,6-BP, the *K*_0.5_ for PEP^3-^ was 0.069 ± 0.004 and 0.060 ± 0.004 for the His_6-_tagged and non His_6-_tagged protein, respectively. The *V*_max_ was 40% of that of the enzyme with the His_6_ tag. Considering that *Vc*IPK is unstable, this difference may be due to the longer purification procedure of the enzyme. Also, the presence of contamination with other proteins, the most abundant being the tryptophanase of *E*. *coli* (identified by sequencing protein fragments by mass spectrometry, data not shown), can explain the lower *V*_max_. Tryptophanase has similar molecular weight and pI as those of *Vc*IPK. Therefore, the studies of *Vc*IPK and *Vc*IIPK were performed with the His_6_ tag.

### Preparation of chromium-ADP

Chromium-ADP was prepared according to [18].

### Pyruvate kinase assays

Ammonium sulfate suspensions of lactate dehydrogenase (EC 1.1.1.27) (LDH) were obtained from Roche Applied Science. Desalted enzymes were prepared as described in Kasahara and Penefsky [19]. Reaction mixtures contained less than 10 μM of contaminating NH_4_^+^, Na^+^, and K^+^ were present as indicated in [20]. The formation of pyruvate was measured at 25°C in a coupled system with LDH and NADH [21]. The reaction mixtures contained 50 mM HEPES, pH 7.0 and the concentrations of monovalent cations (Li^+^, Na^+^, K^+^, NH_4_^+^, Rb^+^ and Cs^+^), divalent cations (Mg^2+^ and Mn^2+^), the substrates phosphoenolpyruvate (PEP) and ADP, allosteric modulators Fru 1,6-BP and ribose 5 phosphate (Rib 5-P) and inhibitors (oxalate and ADP-Cr^2+^) as indicated under “Results and Discussion”. The software CHELATOR [22] was used to calculate ADP-Mg complexes and Mg^2+^_free_ concentrations. In order to determine ADP-Mn complexes and Mn^2+^_free_ concentrations, the *K*_*d*_ of Mn^2+^ was used [23]. The ionized PEP (PEP^3-^) concentrations were calculated considering a pK value of 6.3 [24]. To adjust for the different ligand concentrations, tetramethylammonium chloride (TMACl) was used to maintain constant ionic strength. Sufficient quantities of LDH were added to counteract the inhibitory effect caused by oxalate in the inhibition assays. A 5-fold inclusion of LDH did not result in an increase in the specific activities of PK. The experiments were carried out at 25°C and the reaction was initiated with the addition of either *Vc*IPK or *Vc*IIPK.

### Kinetic studies

The initial velocities of *Vc*IPK and *Vc*IIPK activities were determined in absence or presence of dead-end inhibitors (oxalate or ADP-Cr^2+^). In the former condition, the velocity patterns were obtained at various PEP^3-^ concentrations and several fixed concentrations of ADP-Mg or ADP-Mn. In the latter condition, the inhibition patterns were obtained by varying the concentration of one substrate with the second substrate fixed and at different fixed concentrations of the inhibitor. All these experiments were conducted in the presence of Fru 1,6-BP and Rib 5-P for *Vc*IPK and *Vc*IIPK, respectively.

### Fluorescence experiments

Intrinsic fluorescence emission spectra and anisotropy measurements of *Vc*IPK and *Vc*IIPK were performed in an ISS PC1 photon counting spectrofluorometer (ISS, Urbana, IL) at 25°C. Fluorescence was measured at an excitation wavelength of 280 nm and emission was collected from 290 to 400 nm with 8-nm excitation and emission slits in one milliliter cells. Spectra without protein were subtracted from those containing the enzymes. Fluorescence anisotropy determinations of PKs in the presence of various ligands, emission at λ_max_ was recorded upon excitation at 280 nm. Excitation and emission slits were 8-nm.

### Polyclonal anti-*Vc*IPK and anti-*Vc*IIPK antibodies

Peptides from *Vc*IPK and *Vc*IIPK were synthesized by Genescript. (NJ, USA). These peptides comprised from residue 419 to 436 of *Vc*IPK and from residue 427 to 444 of *Vc*IIPK. Both peptides were coupled to keyhole limpet hemocyanin (KLH). 200 μg of each peptide were mixed with 500 μl (phosphate-buffered saline) PBS buffer and 500 μl complete Freund´s adjuvant (SIGMA) to form a stable emulsion. One rabbit was immunized with each antigen by subcutaneous injection in the large muscle of the rear legs with 200 μl of the emulsion. Three weeks later, a blood sample was collected to determine the serum antibody titers, followed by a booster injection with a similar dose of the antigen and the Freund´s incomplete adjuvant. After two more weeks, the animals were bled out and the sera was separated by centrifugation at 4°C at 3000 rpm for 30 minutes without further purification. Aliquots of 50 μl were stored at -70°C.

### Western blots against *Vc*IPK and *Vc*IIPK

*Vibrio cholerae* (strain CVD 103), *E*. *coli* DH5α, and *E*. *coli* PB25 cell extracts (0.5 ml) were diluted 1:20 in a buffer that contained 20 mM Tris-HCl, 10% glycerol, 50 mM sucrose, 1 mM EDTA, 1 mM ATP, 1mM PMSF, 5 mM benzamidine and a cocktail of proteases, Complete (Roche) 4 tablets/l (pH 7.6) and lysed by sonication. Protein concentrations were determined by the Lowry method [25] and separated using denaturing SDS-PAGE overnight at 10°C (Laemmli gels [16] at 8% polyacrylamide) in parallel to purified recombinant *Vc*IPK or *Vc*IIPK. The gels were then transferred to PVDF membranes. Western blots were carried out with anti-*Vc*IPK and anti-*Vc*IIPK polyclonals antibodies at a 1:30,000 dilution factor. Secondary goat anti-rabbit-HRP (Santa Cruz Biotechnology) against primary antibodies and Streptactin-HRP against Western-C standards were mixed at 1:30,000 and 1:120,000 dilutions, respectively. Blots were then developed using the chemiluminescent HRP substrate detection kit of Millipore (Immoblion^™^ Western). An estimation of the amount of expressed *Vc*IPK and *Vc*IIPK was calculated by densitometry using GelQuant software of DNR Bio-Imaging Systems, Ltd.

Monospecificity of anti-*Vc*IPK and anti-*Vc*IIPK was probed against *Vc*IPK, *Vc*IIPK and *Vc*IIIPK. Purified recombinant *V*. *cholerae* enzymes were separated overnight at 10°C (Laemmli gels [16] at 8% polyacrylamide). The gel was transferred to PVDF and the membrane cut in the middle as indicated in the corresponding figure legend. Blots probing anti-*Vc*IPK and anti-*Vc*IIPK were carried out separately with each half-PVDF membrane as described above.

### Determination of metabolites in *Vibrio cholerae* extracts

Bacteria were cultured in 500 ml of LB inoculated with 1 ml of an overnight pre-inoculum culture. The cells were grown overnight at 37°C and 200 rpm. Afterwards, the absorbance at 600 nm was determined by serial dilutions to determine the final number of cells in the culture assuming that A600 of 0.7 = 6 X 10^8^ cells/ml [26]. Cells were harvested by centrifugation at 2,930 × *g* for 15 min at 4°C and washed twice with PBS (pH 7.4). The cell pellet was resuspended in 2–3 ml of PBS and 0.5 ml of a 30% perchloric acid and 10 mM EDTA solution was added and the cell vigorously vortexed to induce lysis. The cell lysate was incubated for 45–60 min on ice and centrifuged at 12,062 × *g* for 10 min. The supernatant was recovered and neutralized by addition of a buffer containing 0.1 M Tris and 3M KOH. The final sample volume was registered and immediately used. Just before metabolite determination, the samples were centrifuged to remove any particulate material.

Metabolites were determined by spectrophotometry at 340 nm following the NADPH production or NADH consumption using coupling enzymatic systems as described in [27]. The initial reaction mix contained 1.74 ml of 50 mM HEPES supplemented with 1 mM EGTA at pH 7.4, 10 mM MgCl_2_, 1 mM NADP^+^ and 10 mM glucose. 0.2–0.5 ml of neutralized cell extracts were added and the basal absorbance in the absence of added enzymes was recorded. Coupling enzymes were sequentially added to determine specific metabolites within the same sample as follows: for glucose 6- phosphate (Glc 6-P), 2 units of glucose 6-phosphate dehydrogenase (EC 1.1.1.49) from *Leuconostoc mesenteroides* (Roche, Mannheim Germany) were added; for fructose 6-phosphate (Fru 6-P), 7 U of glucose 6-phosphate isomerase (EC 5.3.1.9) (Roche) was added; for ATP, 3 U of hexokinase (EC 2.7.1.1) (Roche) were added. Finally, for Fru 1,6-BP, 10 mM Pi and 1 U of recombinant *Entamoeba histolytica* pyrophosphate-dependent phosphofructokinase (EC 2.7.1.90) [28] were also added. From the stepwise increase in NADPH formation, the metabolite content was determined. Rib 5-P was determined in a 1 ml final volume reaction. The reaction mixture contained 0.9 ml of 100 mM Tris pH 7.0, 0.15 mM NADH and 0.1–0.2 ml of neutralized and clarified extract. Afterwards, 3.4 U glycerol-3 phosphate dehydrogenase (EC 1.1.1.94) (Roche) were added, followed by 100 U triosephosphate isomerase (EC 5.3.1.1) (SIGMA). Further 0.7 mM D-xylulose-5-phosphate (Xul 5-P) lithium salt (SIGMA), 1 mM thiamine pyrophosphate and 10–20 U *Saccharomyces cerevisiae* transketolase (EC 2.2.1.1) (TK) (SIGMA) were also added. The formation of glyceraldehyde-3-phosphate (G 3-P) from Rib 5-P and Xul 5-P through TK was determined. Commercial Xul 5-P was contaminated with Rib 5-P, hence an assay was run in parallel, in the absence of cell sample and using different amounts of Xul 5-P. With this latter assay, it was determined that Xul 5-P contained 40–50% Rib 5-P, but not erythrose-4-phosphate (Ery 4-P). Therefore, for Rib 5-P determination this contamination was subtracted. Furthermore, TK can also form G 3-P and Fru 6-P from Xul 5-P and Ery 4-P, the latter may also be present in the cell sample. Therefore, another assay was prepared in parallel containing 0.8 ml 100 mM Tris-HCl pH 7.0, 1 mM NADP^+^, 0.2 ml of clarified extract, 7 U glucose-6-phosphate isomerase, 2 U glucose-6-phosphate dehydrogenase, 0.2 mM commercial Xul 5-P, 1 mM thiamine pyrophosphate and 10–20 U TK. The change in absorbance was minimal. For the purpose of ensuring the control of the enzymatic assay, commercial Ery 4-P was added, obtaining a high increase in NADPH production. This result indicated that the amount of Ery 4-P in the cells was below the limit of detection of the assay. The intracellular volume of *V*. *cholerae* was determined using the width and length values of the cell [29] using the equation described in [30]. The product of the intracellular volume multiplied by the number of cells (6 x 10^8^ cells/ml) at an A_600nm_ = 0.7 was used to obtain the total intracellular volume of the cell sample. This value was used to determine the concentration of the metabolites.

## Results and discussion

The *Vibrio cholerae* genome contains two circular chromosomes [1], and genes *pyk-F* and *pyk-A-1* are in chromosome 1, whereas *pyk-A-2* is in chromosome 2 [2]. The products of these genes are referred as *Vc*IPK, *Vc*IIPK and *Vc*IIIPK (not studied), respectively. Duplication of pyruvate kinase genes (PK) is frequently found in various γ-proteobacteria [13, 31]. It is of interest to understand if these enzymes are expresssed under common culture conditions (LB and aerobiosis), and if the optimal conditions to express their activity are found in the bacterium. Since no information is available in the literature about PKs from *V*. *cholerae*, the kinetic characterization of the isolated enzymes is described here.

### Purification of *Vc*IPK and *Vc*IIPK and molecular mass determination

**Fig 1:**
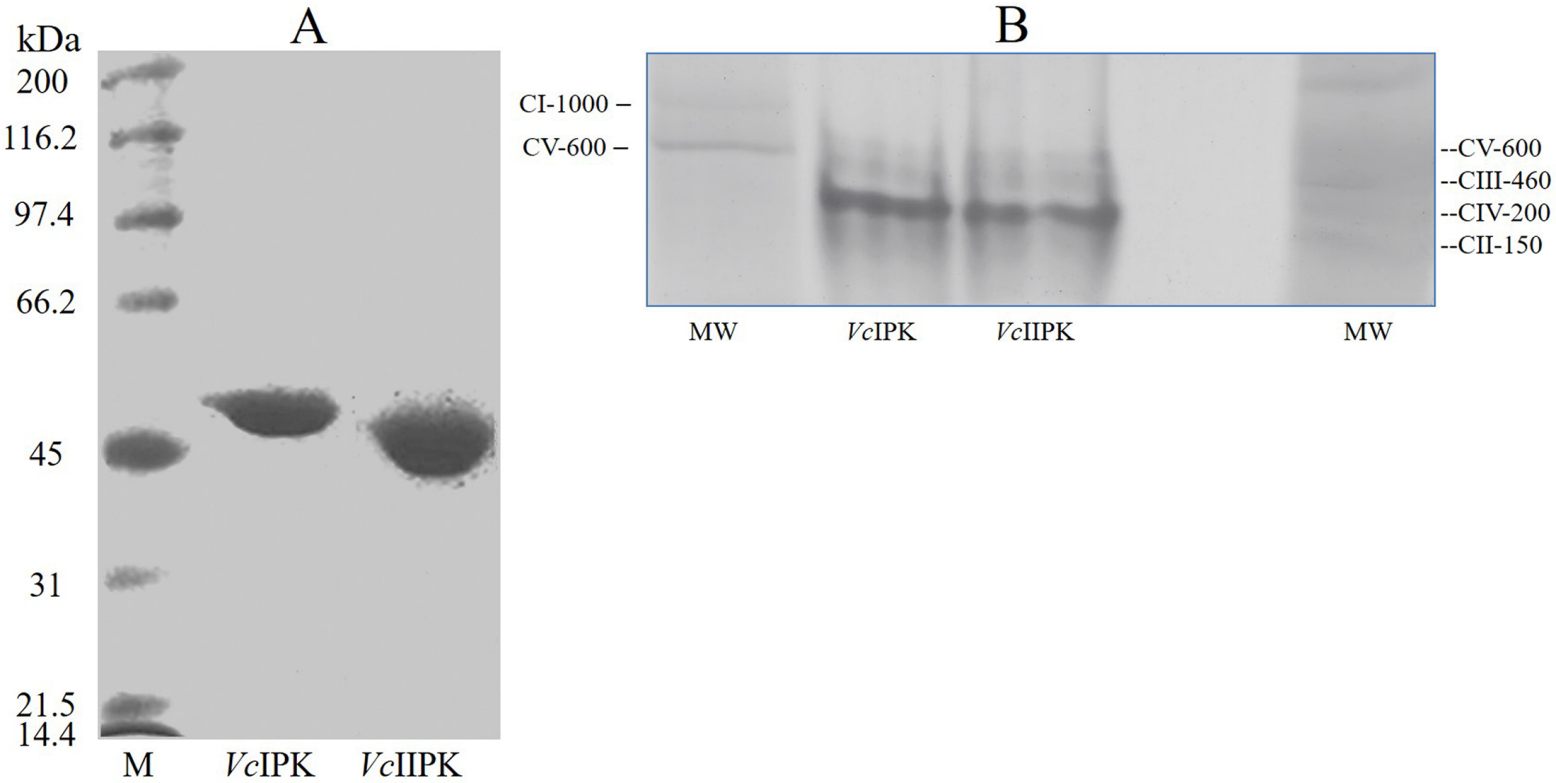
SDS PAGE (12.5%) (A) and blue native PAGE (4–11%) (B) of *Vc*IPK and *Vc*IIPK. In (A) M indicates molecular weight standards (broad range markers BIO-RAD). Amounts of protein loaded were: 15 μg of *Vc*IPK and 20 μg of *Vc*IIPK. In (B) 100 μg of solubilized extract of lauryl maltoside inverted membranes of *Paracoccus denitrificans* (lane 1) and 100 μg of mitochondrial extract from rat liver (lane 5) were used as molecular weight markers. 50 μg of *Vc*IPK (lane 2) and *Vc*IIPK (lane 3) were loaded, respectively. Lane 4 was left empty. Protein staining was performed with Coomassie Blue R-250.

*Vc*IPK and *Vc*IIPK were purified as described in Material and Methods. SDS PAGE revealed a single band of approximately 50 kDa, proving its high degree of purity (Fig 1A). BN-PAGE [16] was used to determine the oligomeric state of both enzymes. As in most PKs [32], a native band of approximately 200 kDa was observed, indicating that both isozymes are homotetramers (Fig 1B). Mass spectrometry of the *Vc*IPK and *Vc*IIPK yielded a molecular weight of 53,138 Da and 54,914 Da, respectively; 50,513 Da and 52,289 Da correspond to the monomers of *Vc*IPK and *Vc*IIPK, respectively when taking the size of 2,624.7 Da corresponding to the His_6_ tag and the cleavage site of protease TEV (data not shown). The calculated molecular weights (protparam Expasy tools) of *Vc*IPK and *Vc*IIPK were, in concordance to mass spectrometry, 50,439 Da and 52,333 Da, respectively.

### Monovalent cation activation of *Vc*IPK and *Vc*IIPK

**Fig 2:**
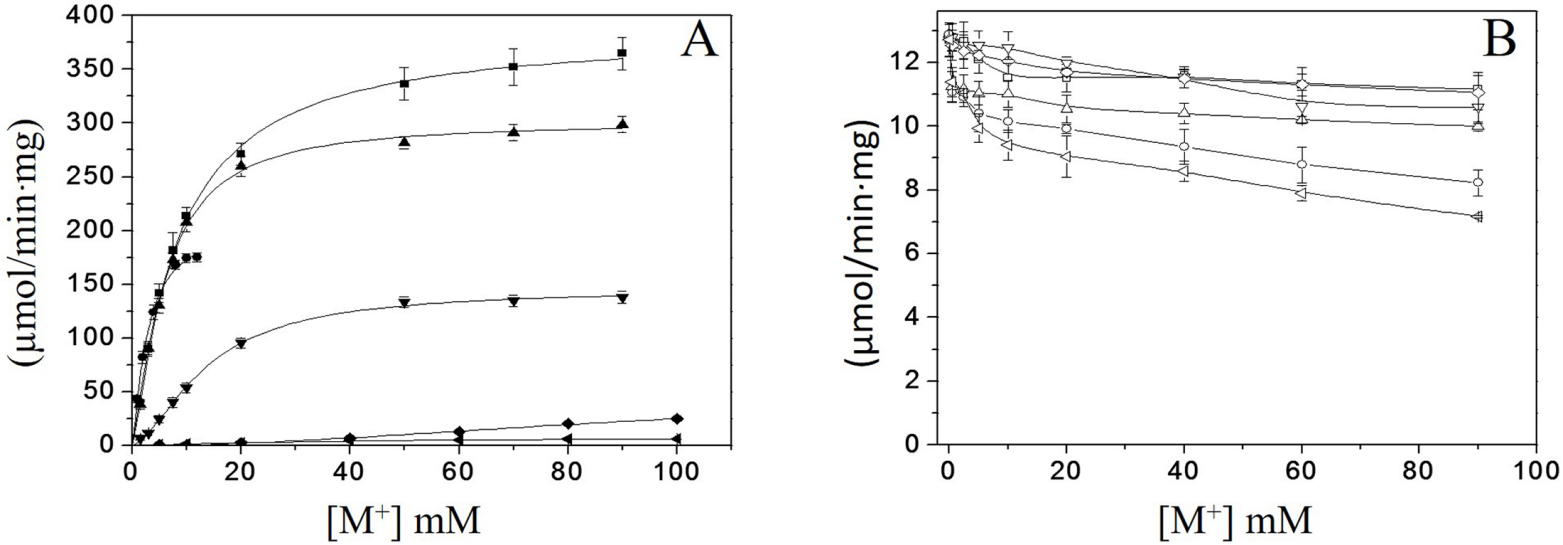
Effect of monovalent cations on *Vc*IPK (A) and *Vc*IIPK (B) activities. *Vc*IPK (closed symbols) and *Vc*IIPK (open symbols) activities in the presence of K^+^ (■,□), Rb^+^ (▲,△), NH_4_^+^ (●,○), Cs^+^ (▼,▽), Na^+^ (◆, ◇) or Li^+^ (◀,◁) are shown. The reaction mixtures contained 25 mM HEPES pH 7.0, 0.2 mM NADH, 8 μg/ml LDH and TMACl in order to maintain the ionic strength at 300 and 450 mM in *Vc*IPK and *Vc*IIPK, respectively. In (A) the concentrations of PEP^3-^, ADP-Mg and Mg^2+^_free_ were 16 mM, 6.5 mM and 2 mM, respectively. In (B) the experiments were carried out with 30.8 mM PEP^3-^, 9 mM ADP-Mg and 15 mM Mg^2+^_free_. Assays were performed at 25°C and the reactions were started by the addition of PK. Amounts of PK ranged from 0.1 to 0.5 μg/ml. The data of figure 2A were fitted to the Hill equation. Standard deviation bars of three experiments are shown.

In a previous phylogenetic study from the family of pyruvate kinase [3], it was generalized that those PKs that have Glu at the position 117 (numbered according to RMPK) exhibited a K^+^-dependent activity, whereas those with Lys in that position have been demonstrated to have a K^+^-independent activity. In agreement with the phylogenetic study [3] *Vc*IPK containing Glu 117 (Fig 2A) and *Vc*IIPK containing Lys 117 (Fig 2B) exhibited K^+^-dependent and K^+^-independent activity, respectively. The activation of *Vc*IPK and *Vc*IIPK by different concentrations of Li^+^, Na^+^, K^+^, NH_4_^+^, Rb^+^ and Cs^+^ (Fig 2) was determined in media that contained a ten-fold greater concentration of the calculated *K*_*0*.*5*_ for PEP^3-^, ADP-Mg complex and Mg^2+^_free_ of *Vc*IPK. In the case of *Vc*IIPK, due to the lack of saturation for PEP^3-^, the experiments were conducted at the highest substrate concentrations allowed (40 mM of total PEP). In agreement with other PKs [33, 34], *Vc*IPK reached maximal activation in the presence of K^+^ (100%) followed by Rb^+^ (81%), NH_4_^+^ (48%), Cs^+^ (38%), Na^+^ (7%) and Li^+^ (2%) (Fig 2A).

It is important to note that the assay with NH_4_^+^ was performed from 0 to 12 mM because at higher concentrations an inhibitory effect was observed. On the other hand, Na^+^ and Li^+^ up to 100 mM did not exhibit a saturation curve, which is necessary for the calculation of kinetic parameters.

Consistent with the PKs that possess Lys in the position 117 [11, 35–39], *Vc*IIPK exhibited a lack of activation by monovalent cations and a minor inhibitory effect (Fig 2B).

The kinetic parameters for the activation of *Vc*IPK by K^+^, NH_4_^+^, Rb^+^ and Cs^+^ are shown in Table 1, but kinetic parameters for Na^+^ and Li^+^ could not to be calculated, because the lack of saturation to include in Table 1. In comparison with other K^+^-dependent PKs, *Vc*IPK exhibited higher *k*_*cat*_ and 2 to 5-fold lower *K*_0.5_ for the monovalent cations [34, 40]. According to [41,42], VCIPK is a good example of Type Ib activation by monovalent cations. In such a mechanism, M^+^ coordination is absolutely required for catalysis, where both *k*_*cat*_ and *k*_*cat*_*/K*_*m*_ increase hyperbolically with [K^+^] (S1 Fig).

**Table 1:** Kinetic parameters for the monovalent cations in *Vc*IPK at saturating concentrations of PEP^3−^, ADP-Mg complex and Mg^2+^_free_. The data from Fig 2A were fitted to the Hill equation *v* = *V*_max_* [S]^*n*^+*K*_*0*.*5*_^*n*^*+* [S] ^*n*^ (Origin version 7.0). The mean and standard deviation of three experiments are shown.

M^+^	*K*_*0*.*5*_ (mM)*K*_0.5_(mM)	*k*_*cat*_ (s^-1^)	*n*
**K**^**+**^	8.5 ± 0.5	1361 ± 30	1.1 ± 0.1
**Rb**^**+**^	5.9 ± 0.2	1067 ± 13	1.4 ± 0.1
**NH**_**4**_^**+**^	2.7 ± 0.3	723 ± 38	1.3 ± 0.1
**Cs**^**+**^	13.5 ± 0.5	515 ± 8	1.6 ± 0.1

### Activation of *Vc*IPK and *Vc*IIPK by allosteric effectors

**Fig 3:**
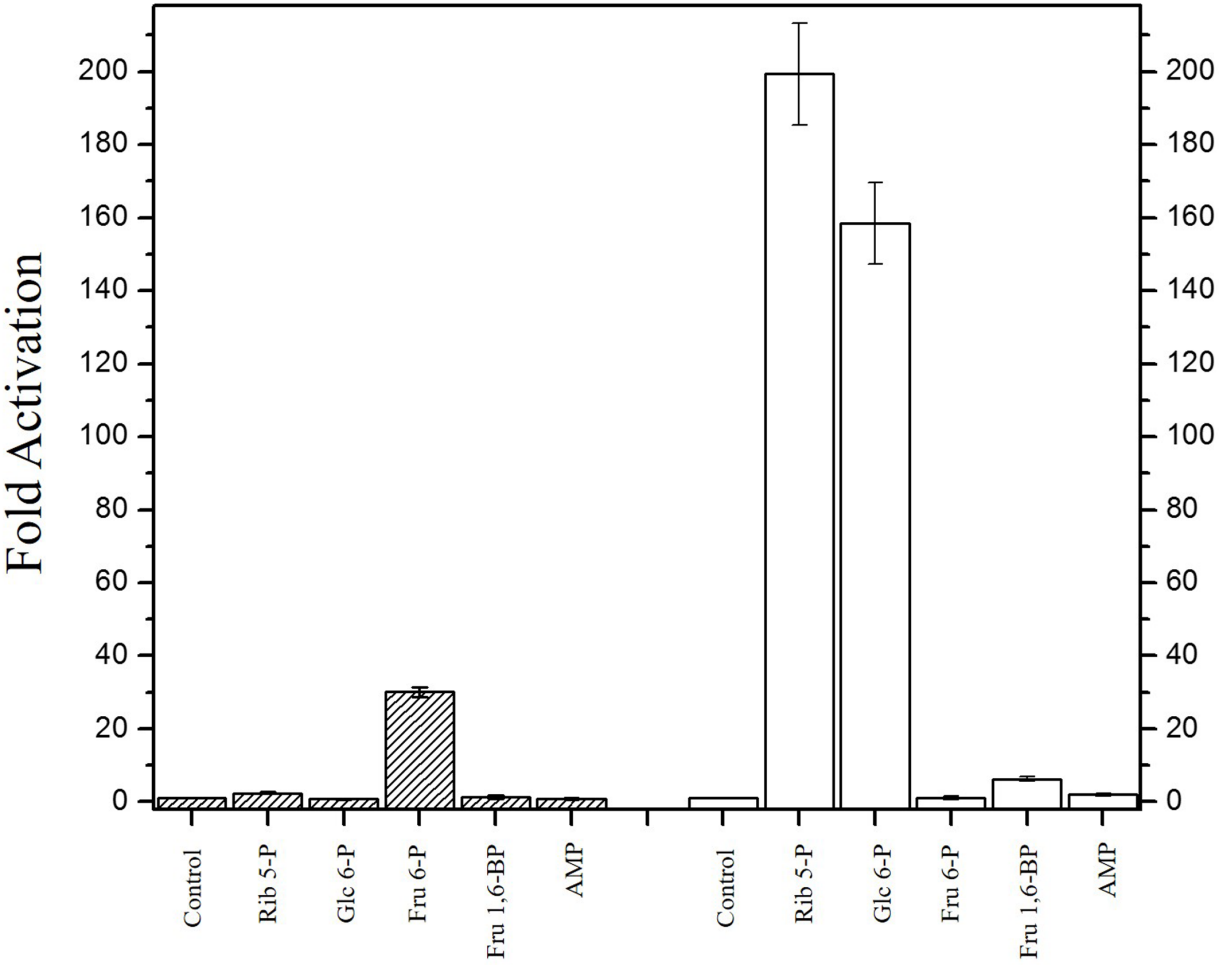
Activation of *Vc*IPK and *Vc*IIPK in the presence of 5 mM of different allosteric modulators. The experimental conditions were 0.5 mM PEP, 6.5 mM ADP-Mg complex and 2 mM Mg^2+^ for *Vc*IPK (dashed bars) and 5 mM PEP, 6.5 mM ADP-Mg complex and 15 mM Mg^2+^ for *Vc*IIPK (open bars). The lanes without or with the respective allosteric effector were indicated: control (in the absence of allosteric effector), Rib 5-P, Glc 6-P, Fru 6-P, Fru 1,6-BP and AMP. The activities of the controls were 9 ± 0.12 μmol/min.mg and 1.5 ± 0.11 μmol/min.mg for *Vc*IPK and *Vc*IIPK, respectively. Assays were performed at pH 7.0, 300 mM ionic strength, and at 25°C. The reaction was started by addition of 5 μg/ml PK. Standard deviation bars of three experiments are shown.

It has been reported that K^+^-dependent PKs are allosterically activated by bi-phosphorylated sugars [32] such as Fru 1,6-BP [7, 43] and Fru 2,6-BP [44], and K^+^-independent PKs are mainly activated by AMP [13] and monophosphorylated sugars, such as Glc 6-P [45] and Rib 5-P [12]. In order to express maximal activities of both enzymes, various allosteric effectors of PKs were tested. The experiments with *Vc*IPK were conducted at 0.3 mM of PEP^3-^, a concentration that corresponds to 0.18-times the *K*_*0*.*5*_ in the absence of Fru 1,6-BP, and at a concentration that corresponds to the *K*_*0*.*5*_ obtained for the saturation curve of the substrate in the presence of 5 mM of the modulator (for the latter data, see Table 2). Due to the low activity that *Vc*IIPK exhibited in the absence of the allosteric effector, the assays were performed with 3.61 mM PEP^3-^, 6.5 mM ADP-Mg complex and 30 mM Mg^2+^_free_ where a reliable activity could be measured. Fig 3 shows that *Vc*IPK was activated 31-fold by Fru 1,6-BP (from 9 to 279 μmol/min.mg), whereas *Vc*IIPK was activated 200-fold (from 1.5 to 300 μmol/min.mg) by Rib 5-P and 159-fold (1.5 to 239 μmol/min.mg) by Glc 6-P. However, Rib 5-P is a better allosteric activator than Glc 6-P. In the presence of Glc 6-P, *Vc*IIPK exhibited a *V*_max_ of 229 ± 4 μmol/min.mg, a *K*_*0*.*5*_ for PEP^3-^ of 1 ± 0.02 mM and a Hill number of 2.4 ± 0.1. These data are 12% lower, 3-fold higher and 1.3-fold higher than those obtained with 5 mM of Rib 5-P, respectively (compared to the data for PEP^3-^ of *Vc*IIPK in Table 2).

**Table 2:** Kinetic parameters for PEP3-, ADP-Mg and Mg^2+^_free_ in the absence or presence of 0.05 and 5 mM Fru 1,6-BP for *Vc*IPK or of 0.05 and 5 mM Rib 5-P for *Vc*IIPK. The data of Fig 4 were fitted (nonlinear regression Origin version 7.0) to the Hill equation *v* = *V*_max_ * [S]^*n*^+*K*_*0*.*5*_^*n*^*+* [S] ^*n*^. The mean and standard deviation of three to four experiments are shown. N. D. not determined.

Effector (mM)	*Vc*I			*Vc*II		
	*K*_0.5_ (mM)	*k*_*cat*_(s^-1^)	*n*	*K*_0.5_ (mM)	*k*_*cat*_(s^-1^)	*n*
**PEP**^**3-**^						
**0**	1.7 ± 0.04	1114 ± 21	3.7 ± 0.2	N.D.	N.D.	N.D.
**0.05**	0.9± 0.02	1205 ± 14	3.6 ± 0.2	1.7 ± 0.03	792 ± 12	2.9 ± 0.1
**5**	0.3 ± 0.02	1402 ± 29	1.0 ± 0.1	0.3 ± 0.01	959 ± 14	1.8 ± 0.1
**ADP-Mg**						
**0**	0.6 ± 0.02	1294 ± 18	1.0 ± 0.03	2.3 ± 0.3	145 ± 8	1.1 ± 0.1
**0.05**	0.4 ± 0.02	1348 ± 20	1.2 ± 0.1	0.4 ± 0.03	836 ± 18	1.2 ± 0.1
**5**	0.5 ± 0.02	1313 ± 19	1.3 ± 0.1	0.3 ± 0.02	971 ± 21	1.1 ± 0.1
**Mg**^**2+**^						
**0**	0.2 ± 0.01	1228 ± 15	1.5 ± 0.1	33 ± 6	206 ± 22	1.1 ± 0.1
**0.05**	0.3 ± 0.02	1260 ± 31	1.4 ± 0.1	2.0 ± 0.1	814 ± 17	2.3 ± 0.2
**5**	0.3 ± 0.01	1282 ±21	1.4 ± 0.1	1.4 ± 0.03	849 ± 11	2.9 ± 0.2

### Assays of *Vc*IPK and *Vc*IIPK in the presence of Mg^2+^ and Mn^2+^

PKs exhibit an absolute dependence on divalent cations for activity, because they are essential for phosphate transfer [46]. Usually Mg^2+^ or Mn^2+^ are used and may be exchanged due to their similarities in the chelate structures of these ions [47]. However, Mg^2+^ is generally the most efficient physiological cation for PKs, with notable exception of *Corynebacterium glutamicum* PK, that has a preferential use of Mn^2+^ or Co^2+^ [38], *Bradyrhizobium japonicum* PK which only uses Mn^2+^ [48], and *Sporolactobacillus inulinus* PK [49] which strongly prefers Mn^2+^ over Mg^2+^ for activity. Thus, the effects of Mg^2+^and Mn^2+^on the activity of *Vc*IPK and *Vc*IIPK were studied.

#### Kinetic parameters for *Vc*IPK and *Vc*IIPK in the presence of Mg^2+^

**Fig 4:**
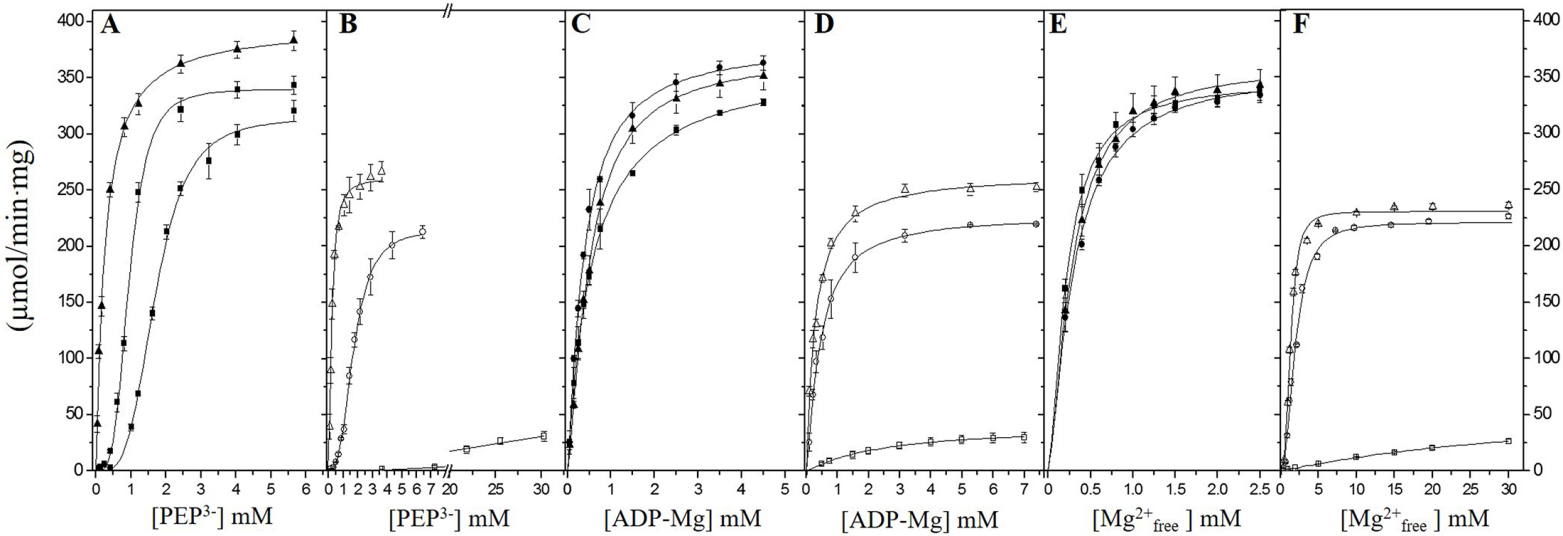
The effect of Fru 1,6-BP in *Vc*IPK and Rib 5-P in *Vc*IIPK on the saturation curves for PEP^3-^, ADP-Mg and Mg^2+^_free_. In (A, C and E) the effect of Fru 1,6-BP (closed symbols) is shown in *Vc*IPK and in (B, D and F) the effect of Rib 5-P (open symbols) is shown in *Vc*IIPK. The concentrations of the allosteric activators were (■,□) 0 mM, (●,○) 0.05 mM and (▲,△) 5 mM. The reaction mixtures contained 25 mM HEPES pH 7.0, 0.2 mM NADH, 8 μg/ml LDH, TMACl in order to maintain the ionic strength at 300–350 mM and with and without 90 mM KCl in *Vc*IPK and *Vc*IIPK, respectively. Kinetics for PEP^3-^ and ADP-Mg were performed under saturating concentrations of the other substrate. The concentrations of Mg^2+^_free_ for the saturation curves of PEP^3-^ and ADP-Mg were 2 mM and 15 mM for *Vc*IPK and *Vc*IIPK, respectively. For the saturation curves of Mg^2+^_free_, the concentration of PEP was varied from 5 to 40 mM and those of ADP-Mg from 3 to 9 mM. Assays were performed at 25°C and the reactions were started by the addition of 0.1 to 5 μg/ml of PK. The data were fitted to the Hill equation. Standard deviation bars of three to four experiments are shown.

Saturation curves for PEP^3-^ (Fig 4A and 4B), ADP-Mg (Fig 4C and 4D) and Mg^2+^_free_ (Fig 4E and 4F) of *Vc*IPK and *Vc*IIPK are shown, respectively. All experiments were conducted in the absence or presence of 50 μM or 5 mM of Fru 1,6-BP or Rib 5-P for *Vc*IPK and *Vc*IIPK, respectively. As shown in Fig 4A, 4C and 4E, *Vc*IPK exhibited high activity with or without the effector indicating that Fru 1,6-BP was a non-essential activator. As in other PKs [13], Fru 1,6-BP activated *Vc*IPK by decreasing the *K*_*0*.*5*_ for PEP^3-^ with minor or no effect on the kinetic constants for ADP-Mg complex or Mg^2+^_free_. The allosteric activator, decreased the *K*_*0*.*5*_ for PEP^3-^ by seven-fold, increased the *k*_*cat*_ by 20%, and decreased the Hill number from 3.7 to 1 (Fig 4A and Table 2). On the other hand, Fig 4B, 4D and 4F show that *Vc*IIPK in the presence of non-physiolologically high concentrations of the substrates (40 mM PEP, 39 mM Mg^2+^ and 9.7 mM ADP) exhibited low activities (25 μmol/min.mg) in the absence of the Rib 5-P. Even at the highest PEP concentration tested no saturation kinetics was observed without the allosteric effector. In contrast, in the presence of Rib 5-P, a saturation curve for PEP^3-^ in *Vc*IIPK was observed indicating that Rib 5-P was an essential activator. The allosteric effector also decreased the *K*_*0*.*5*_ for all the substrates (PEP^3-^, ADP-Mg and Mg^2+^_free_) by at least one order of magnitude, increased the *k*_*cat*_ in the range of 400 to 700% and varied the Hill number in a differential manner for each substrate (Table 2). Moreover if the low substrate concentrations (0.091–0.300 mM PEP, 0.55–0.823 mM ADP and 1–3 mM Mg^2+^_free_) found *in vivo* in other γ –proteobacteria [50–53] were used in the assays, the activity of *Vc*IIPK would absolutely rely on Rib 5-P. This allosteric regulation is particular for this PK.

#### Kinetic parameters for *Vc*IPK and *Vc*IIPK in the presence of Mn^2*+*^

**Fig 5:**
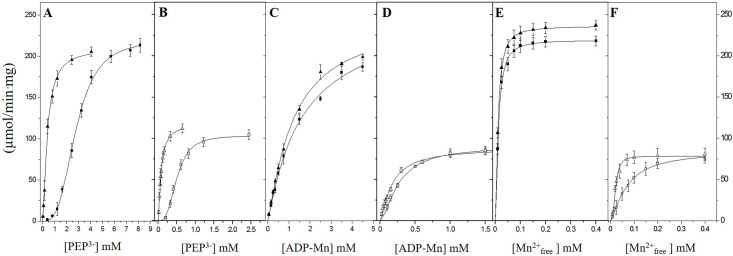
The effect of Fru 1,6-BP in *Vc*IPK and Rib 5-P in *Vc*IIPK on the saturation curves for PEP^3-^, ADP-Mn and Mn^2+^_free_. In (A, C and E) the effect of Fru 1,6-BP (closed symbols) is shown in *Vc*IPK and in (B, D and F) the effect of Rib 5-P (open symbols) is shown in *Vc*IIPK. The concentrations of the allosteric activators were (■,□) 0 mM and (▲,△) 5 mM. The reaction mixtures contained 25 mM HEPES pH 7.0, 0.2 mM NADH, 8 μg/ml LDH, and TMACl in order to maintain the ionic strength at 300 mM, and also with and without 90 mM KCl in *Vc*IPK and *Vc*IIPK, respectively. Kinetics for PEP^3-^ and ADP-Mg were performed at saturating concentration of the other substrate. The concentrations of Mn^2+^_free_ on the kinetics for PEP^3-^ and ADP-Mn were 0.5 mM for *Vc*IPK and 0.35 mM for *Vc*IIPK. For the saturation curves of Mn^2+^_free_, the concentrations of PEP were 20 mM and 11 mM for *Vc*IPK and *Vc*IIPK, respectively. The concentration of ADP-Mn was 3 mM for both enzymes. Assays were performed at 25°C and the reaction was started by addition of 0.1 to 0.5 μg/ml of PK. The data were fitted to the Hill equation. Standard deviation bars of three to four experiments are shown.

Saturation curves for PEP^3-^ (Fig 5A and 5B), ADP-Mn (Fig 5C and 5D) and Mn^2+^_free_ (Fig 5E and 5F) of *Vc*IPK and *Vc*IIPK are shown, respectively. All the experiments were conducted in the absence or presence of 5 mM of Fru 1,6-BP or of 5 mM Rib 5-P for *Vc*IPK and *Vc*IIPK, respectively. *Vc*IPK exhibited lower activities in the presence of Mn^2+^ (Fig 5A, 5C and 5E) compared to those in the presence of Mg^2+^ (Fig 4A, 4C and 4E) either with or without Fru 1,6-BP. This result is in agreement with other PKs [15, 46, 54, 55]. Although the maximum activity for *Vc*IPK was 1.4-fold higher with Mg^2+^ than with Mn^2+^, the *K*_*0*.*5*_ for Mn^2+^_free_ was 17-fold lower than the *K*_*0*.*5*_ for Mg^2+^_free_, with no significant change in the constants for the PEP^3-^ and ADP-Mn (see Tables 2 and 3). In agreement with the results in the presence of Mg^2+^, Fru 1,6-BP activated *Vc*IPK by decreasing the *K*_*0*.*5*_ for PEP^3-^ by 7-fold (Fig 5A) with minor or no effect on the kinetic constants for ADP-Mn complex or Mn^2+^_free_ (Fig 5C and 5E)_._ Remarkably, in the absence of Rib 5-P and presence of Mn^2+^, *Vc*IIPK exhibits a saturation curve for PEP^3-^ (Fig 5B). This is in contrast to the non-saturation kinetics observed in the presence of Mg^2+^ (Fig 4B). In these experimental conditions, it is relevant to mention, that *Vc*IIPK with Mn^2+^ exhibits 2-fold higher activities than those with Mg^2+^ for PEP^3-^ and ADP-Mn (Figs 5B, 5D and 5F *vs*. 4B, 4D and 4F). In addition, the *K*_*0*.*5*_ for Mn^2+^ and ADP-Mn was 475-fold and 9-fold lower than that for Mg^2+^ and ADP-Mg, respectively (see Tables 2 and 3). Although the kinetic parameters for PEP^3-^ of *Vc*IIPK could not be determined with Mg^2+^, the *K*_*0*.*5*_ for PEP^3-^ with Mn^2+^ is at least one order of magnitude lower than that with Mg^2+^ (Figs 4B *vs*. 5B). In contrast to the requirement for Rib 5-P of *Vc*IIPK to express catalysis when the divalent cation is Mg^2+^, in the presence of Mn^2+^ similar *V*_max_ of the enzyme were attained with or without the allosteric effector. Rib 5-P activated *Vc*IIPK by decreasing the *K*_*0*.*5*_ for PEP^3-^ by 7-fold with minor (3-fold) or no effect on the kinetic constants for Mn^2+^_free_ or ADP-Mn complex, respectively. These results suggest that the interaction of Mn^2+^ with *Vc*IIPK is different than that of Mg^2+^, and that Mn^2+^ may “mimic” the allosteric effect of Rib 5-P, as stated in yeast PK with Fru 1,6-BP [56]. In agreement with this finding, the role of Fru 1,6-BP has been questioned in the presence of Mn^2+^ in other PKs [48, 56, 57].

**Table 3:** Kinetic parameters for PEP^3-^, ADP-Mn and Mn^2+^_free_ in the absence or presence of 5 mM Fru 1,6-BP or of 5 mM Rib 5-P for *Vc*IPK and *Vc*IIPK, respectively. The data of Fig 5 were fitted (nonlinear regression Origin versión 7.0) to the Hill equation *v* = *V*_max_* [S]^*n*^+*K*_*0*.*5*_^*n*^*+* [S] ^*n*^. The mean and standard deviation of three to four experiments are shown.

Effector (mM)	*Vc*I			*Vc*II		
	*K*_0.5_ (mM)	*k*_*cat*_(s^-1^)	*n*	*K*_0.5_ (mM)	*k*_*cat*_(s^-1^)	*n*
**PEP**^**3-**^						
**0**	2.8 ± 0.1	778 ±11	3.1 ± 0.2	0.5 ± 0.01	381 ± 9	3 ± 0.2
**5**	0.4 ± 0.03	740 ± 21	1.5 ± 0.1	0.08 ± 0.003	435 ± 9	1.3 ± 0.1
**ADP-Mn**						
**0**	1.7 ± 0.3	919 ± 71	1.0 ± 0.1	0.3 ± 0.01	333 ± 3	1.5 ± 0.1
**5**	1.2 ± 0.2	876 ± 48	1.2 ± 0.1	0.2 ± 0.02	348 ± 14	1.1 ± 0.1
**Mn**^**2+**^						
**0**	0.01 ± 0.0004	776 ± 7	1.6 ± 0.1	0.07 ± 0.01	310 ± 11	1.4 ± 0.1
**5**	0.01 ± 0.0002	836 ± 6	1.4 ± 0.1	0.02 ± 0.001	287 ± 11	2.9 ± 0.04

### Initial velocity studies of *Vc*IPK and *Vc*IIPK

**Fig 6:**
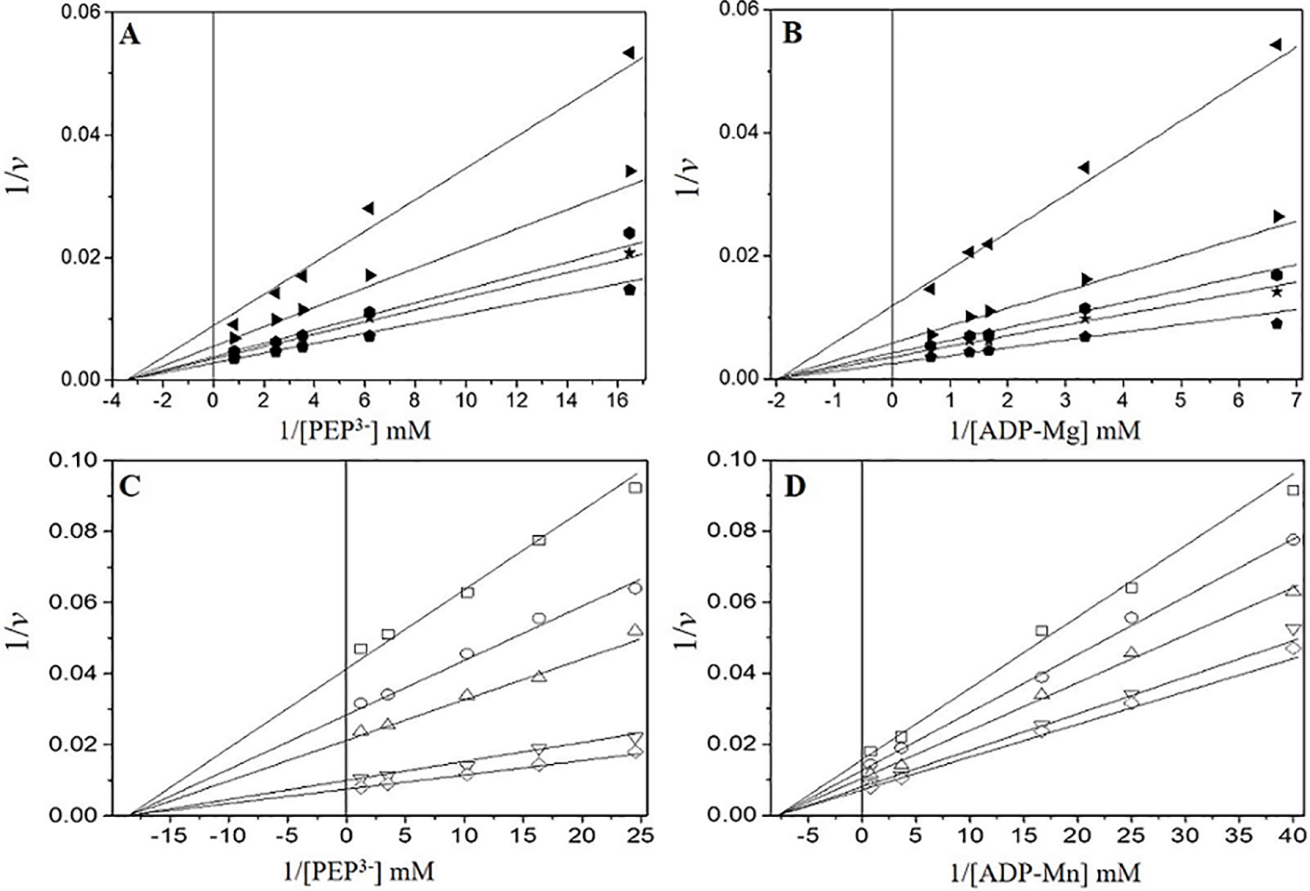
Double reciprocal plots from the initial velocity data of the reaction catalyzed by *Vc*IPK (A and B) and *Vc*IIPK (C and D). The reaction mixtures contained 50 mM HEPES pH 7.0, 0.2 mM NADH, 8 μg/ml LDH, 300 mM of ionic strength (TMACl), with 90 mM of KCl and 5 mM of Fru 1,6-BP in *Vc*IPK and without KCl and 5 mM of Rib 5-P in *Vc*IIPK. The reciprocals of the concentrations of PEP^3-^ and ADP-M^2+^ complexes are shown in the abscissas of each graph. The variable fixed concentrations of ADP-Mg in (A) were 0.15 (◀), 0.3 (►), 0.6 (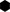), 0.75 (★) and 1.5 mM (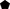) and ADP-Mn in (C) were 0.025 (□), 0.04 (○), 0.06 (△), 0.275 (▽) and 1.3 mM (◇). The variable fixed concentrations of PEP^3-^ in plot B were 0.06 (◀), 0.16 (►), 0.28 (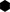), 0.4 (★) and 1.21 mM (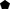) and in plot D were 0.0408 (□), 0.061 (○), 0.097 (△), 0.28 (▽) and 0.81 mM (◇). In (A and B) the Mg^2+^_free_ concentration was kept constant at 2 mM and Mn^2+^_free_ concentration in (C and D) was fixed at 0.35 mM. The reaction was started by the addition of PK, the amounts of PK ranged from 0.1 to 0.5 μg/ml. The fitted data are shown in Table 4.

To date initial velocity studies of PK have been focused on RMPK. This enzyme follows a random order rapid equilibrium kinetic mechanism at saturating concentrations of K^+^, as first demonstrated by Boyer’s group [58], and confirmed later by others [18, 59, 60]. In the absence of K^+^, RMPK changes its kinetic mechanism to ordered rapid equilibrium with PEP being the first substrate [18]. Similar studies have been conducted in the K^+^-dependent PK of the muscle of the sea mollusk *Concholepas concholepas* [61], and in the K^+^-independent PK of the crenarchaeota *Thermophilum pendens* [15]. It was found that both enzymes follow a rapid equilibrium random order kinetic mechanism. However, when the *C*. *concholepas* enzyme was studied in the presence of Mn^2+^ the results demonstrated an ordered sequential mechanism, with ADP-Mn being the first substrate [61]. In this work, initial kinetic studies were carried out both in the presence and absence of K^+^ for *Vc*IPK (Fig 6A and 6B) and *Vc*IIPK (Fig 6C and 6D), respectively. These experiments were performed at various concentrations of one of the substrates and at fixed concentrations of the other; however, the assays for *Vc*IPK were performed in the presence of Mg^2+^ and Fru 1,6-BP, whereas those for *Vc*IIPK were performed with Mn^2+^ and Rib 5-P. The experimental conditions chosen for *Vc*IPK allowed hyperbolic kinetics and maximal activities, whereas those for *Vc*IIPK were chosen because the enzyme showed a sigmoidal response for PEP^3-^ even in the presence of Rib 5-P either with Mg^2+^ or Mn^2+^. However, this cooperative effect was lower with Mn^2+^ (*n*_H_ = 1.3) than with Mg^2+^ (*n*_H_ = 1.8). Additionally, oxalate inhibition was not observed in *Vc*IIPK in the presence of Mg^2+^.

In *Vc*IPK (Fig 6A) and *Vc*IIPK (Fig 6C), the double reciprocal plots of the initial velocities *versus* the ionized PEP concentrations intersected on the 1/S axis and to the left on the 1/*v* axis. When the concentrations of ADP-Mg in *Vc*IPK (Fig 6B) or ADP-Mn in *Vc*IIPK (Fig 6D) were varied, the lines intersected on the 1/S axis and to the left on the 1/*v* axis. These results indicate either an ordered steady state or a rapid equilibrium random-order kinetic mechanism. These data were globally fitted to the equation described in Table 4, and the kinetic constants obtained are shown. It is observed that both isozymes in the presence of their respective allosteric effectors exhibit similar catalytic efficiencies (Table 4).

**Table 4:** Intersecting patterns, kinetic mechanisms and kinetic constants of *Vc*IPK and *Vc*IIPK. Intersecting patterns were taken from the double reciprocal plots of the initial velocity data. The data shown in Fig 6 were globally fitted (nonlinear regression Origin versión 7.0) to the equation describing a rapid equilibrium random order mechanism *v* = *V*_max_ [A] [B]/ (*K*_*a*_*K*_*b*_ + *K*_*a*_ [B] + *K*_*b*_ [A] + [A] [B]), where *v* represents the initial velocity, A is PEP^3-^, B is ADP-M^2+^ and *K*_*a*_ *and K*_*b*_ are the Michaelis-Menten constants for PEP and ADP-M^2+^, respectively. Standard deviation values are shown. Catalytic efficiency values, *k*_*cat*_*/K*_*m*_ (M^1^s^-1^) are expressed in log form.

Condition	Initial velocity intersectingm patterns		Kinetic mechanism	*V*_*max*_	*K*_*m*_ PEP	*K*_*m*_ *ADP-M*^*2+*^	*k*_*cat*_	Log *k*_*cat*_*/*K_m PEP_	Log *k*_*cat*_*/*K_m ADP-M_^2+^
				μmol min^-1^mg^-1^	mM	mM	s^-1^		
***Vc*IPK with Mg**^**2+**^	Intersects to the left of the 1/*v* axis and on the 1/S axis	Intersects to the left of the 1/*v* axis and on the 1/S axis	Random rapid equilibium	476±20	0.3±0.02	0.5±0.04	1684	6.76	6.54
***Vc*IIPK with Mn**^**2+**^	Intersects to the left of the 1/*v* axis and on the 1/S axis	Intersects to the left of the 1/*v* axis and on the 1/S axis	Random rapid equilibium	147±2	0.06±0.02	0.13±0.02	541	6.95	6.62

### Dead-end inhibition studies of *Vc*IPK and *Vc*IIPK

The use of dead-end inhibitors provide a strong advantage in the determination of the kinetic mechanisms of enzymes [62]. In this work, oxalate was used as dead-end analog of PEP [63] and ADP-Cr^2+^ as that of ADP-M^2+^ [64]. In both enzymes, the patterns of oxalate inhibition *versus* PEP and ADP-M^2+^ were competitive and mixed-type with α < 1 (factor affecting *K*_*i*_ for oxalate inhibition *versus* ADP-M^2+^), respectively. This indicates that the enzyme-oxalate binary complex has a higher affinity for ADP-M^2+^ than that of the free enzyme. In *Vc*IPK assays with ADP-Cr^2+^, the inhibition was non-competitive for PEP and competitive for ADP-Mg. *Vc*IIPK followed the same patterns as those of *Vc*IPK, with the exception that a mixed type pattern with α >1 for ADP-Cr^2+^ inhibition *versus* PEP was observed. This was interpreted, that the free enzyme has higher affinity for PEP than the enzyme-ADP-Cr^2+^ binary complex. Collectively, the data for both enzymes show that oxalate is a competitive inhibitor with respect to PEP, whereas ADP-Cr^2+^ is a competitive inhibitor with respect to ADP-M^2+^. These results demonstrate that the analogs and the substrates bind to the same site. If it is considered that oxalate is a mixed type inhibitor with respect to ADP-M^2+^, it may be concluded that oxalate forms a non-productive ternary complex and thereby diminishes the *V*_max_. The same argument holds for ADP-Cr^2+^ with respect to PEP in *Vc*IIPK, whereas in *Vc*IPK, the non-competitive inhibition of ADP-Cr^2+^ *versus* PEP decreases the *V*_max_, without altering the binding of PEP. In all cases, replots of the slopes or intercepts of the double reciprocal plots against inhibitor concentration were linear (not shown). All the data were globally fitted to the equations that describe linear competitive inhibition, linear non-competitive inhibition or linear mixed inhibition. The inhibition patterns and inhibition constants for *Vc*IPK and *Vc*IIPK are shown in Table 5. Therefore the data with dead-end inhibitors suggest that *Vc*IPK and *Vc*IIPK follow a rapid equilibrium random order kinetic mechanism, in spite of having E74 or K75, respectively (corresponding to the E117 in the sequence of RMPK). These results are in agreement with the mechanism reported earlier for RMPK in the presence of K^+^ and in the mutant E117K of RMPK [18].

**Table 5:** Dead-end inhibition patterns and inhibition constants for oxalate and ADP-Cr^2+^ in *Vc*IPK and *Vc*IIPK. Inhibition patterns were taken from the double reciprocal plots of the inhibition experiments. Simple inhibition patterns were confirmed from linear replots of the slopes or intercepts *versus* the inhibitor concentrations (not shown). The inhibition constants were calculated from the fits of the complete data set to the corresponding equations from linear competitive inhibition (C) *v* = *V*_max_ *[S]/(*K*_*m*_(1+[I]/*K*_*i*_)+[S]), linear noncompetitive inhibition (NC), or linear mixed inhibition (MT) *v =* V_max_ *[S]/*K*_*m*_(1+[I]/*K*_*i*_) + [S](1+[I]/α*K*_*i*_)), where α = 1 and α < 1 for NC and MT, respectively; *K*_*i*_ is the inhibition constant.

*V*. *cholerae* enzyme	Dead end analog of PEP: oxalate		Dead end analog of ADP-M^2+^: ADP-Cr^2+^		*Ki* (oxalate) μM	*Ki* (ADP-Cr^2+^) mM
	1/v *versus* 1/PEP, fixed ADP-M^2+^	1/v *versus* 1/ADP-M^2+^, fixed PEP	1/v *versus* 1/PEP, fixed ADP-M^2+^	1/v *versus* 1/ADP-M^2+^, fixed PEP		
***Vc*IPK**	C	MT	NC	C	7.8 ± 0.3	1.1 ± 0.1
***Vc*IIPK**	C	MT	MT	C	71 ± 6	1.3 ± 0.1

### Fluorescence studies of *Vc*IPK and *Vc*IIPK

**Fig 7:**
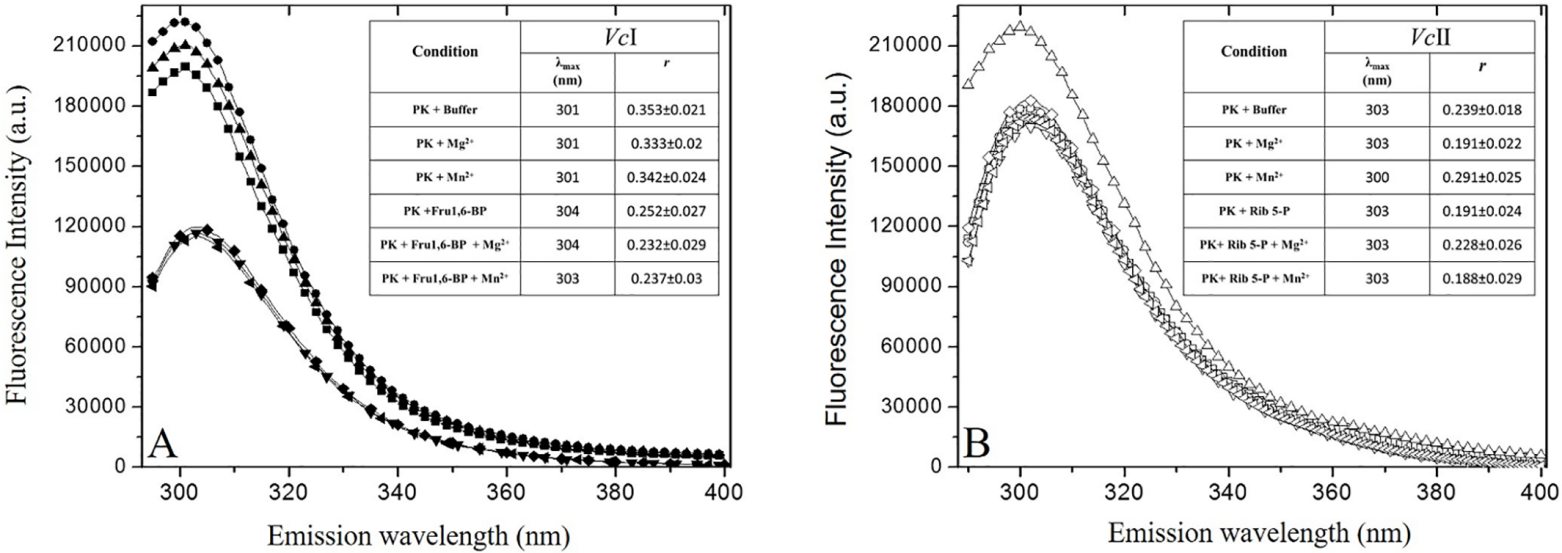
Effect of various ligands on the intrinsic fluorescence emission spectra at 280 nm, λ_max_ and the anisotropy of *Vc*IPK (A) and *Vc*IIPK (B). Closed symbols are for *Vc*IPK and open symbols are for *Vc*IIPK. The symbols represent: PK + HEPES (■,□), PK + Mg^2+^ (●,○), PK + Mn^2+^ (▲,△), PK + Effector (▼,▽), PK + Effector + Mg^2+^ (◆,◇), PK + Effector + Mn^2+^ (◀,◁). Fluorescence studies were performed at 25°C in mixtures that contained 200 μg ml^-1^ of *Vc*IPK or *Vc*IIPK in 50 mM HEPES pH 7.0. In *Vc*IPK, the media contained either 2 mM Mg^2+^, 0.2 mM Mn^2+^ or 5mM of Fru 1,6-BP. In the mixtures that contained Fru 1,6-BP and the divalent cation, the concentration of the effector was 0.5 mM and the concentrations of Mg^2+^ or Mn^2+^ were 2 or 0.2 mM, respectively. In *Vc*IIPK, the media contained either 30 mM Mg^2+^, 0.5 mM Mn^2+^ or 5 mM Rib 5-P. In the mixtures that contained Rib 5-P and Mg^2+^, the concentrations were 1.5 and 15 mM, respectively. When the effector was added with Mn^2+^, the concentration of Rib 5-P was 0.5 and that of the divalent cation was 0.2 mM. The inset shows the λ_max_ and the anisotropy values (*r*). The anisotropy values were calculated at their respective maximal emission.

For fluorescence studies, an excitation at 280 nm for both enzymes was selected due to their lack of tryptophan residues. *Vc*IPK and *Vc*IIPK have 5 and 8 tyrosines per monomer, respectively. Since there is no crystallographic structure reported for these PKs, the distribution of the tyrosines in their sequences, was based on the alignment of the sequences of *E*. *coli* PK type I with *Vc*IPK (78% identity), and on that of the sequences of *E*. *coli* PK type II with *Vc*IIPK (69% identity) [32]. Therefore *Vc*IPK appears to have one Tyr in domain B and two in domain A and C, respectively. *Vc*IIPK appears to harbor one Tyr in domain B, three in domain A and four in domain C. Thus, global conformational changes were determined by tyrosines fluorescence and intrinsic fluorescence anisotropies (*r*) induced by different ligands. As observed in Fig 7A fluorescence intensities, λ_max_ and *r* of *Vc*IPK were similar with or without divalent cations (Mn^2+^ and Mg^2+^). However, the addition of Fru 1,6-BP quenched the intensity 50%, shifted λ_max_ 2–3 nm to the red spectrum, and decreased the *r* of the enzyme in the presence or absence of Mn^2+^or Mg^2+^. In contrast, *Vc*IIPK exhibited no conformational changes in all the experimental conditions assayed, except in the presence of Mn^2+^, where a 20% increase in the fluorescence intensity, a blue shift of 3 nm, and an increase of *r* were observed (Fig 7B). These results indicate that the binding of Fru 1,6-BP to *Vc*IPK caused the tyrosines to become more exposed to the solvent phase, increasing the global protein flexibility. These results are in agreement with the structural effect of Fru 1,6-BP in yeast PK [65, 66] human liver PK [67] and in *E*. *coli* PK [68]. On the other hand, no conformational change induced by its allosteric effector was detected in *Vc*IIPK by fluorescence measurements. Therefore, it might be that tyrosines present in the C domain of this enzyme were not applicable to monitor the movements of the enzyme when R5P was bound.

In the presence of Mn^2+^ the enzyme adopts a more compact and less mobile conformation. This conformation corresponds to its active state as described above in the kinetics of the enzyme with this divalent cation. Likewise, other PKs adopt a compact active conformation induced by ligands in RMPK [18] and by Fru 2,6-BP in trypanosomatid PKs [69, 70].

### Expression of *Vc*IPK and *Vc*IIPK in *Vibrio cholerae*

**Fig 8:**
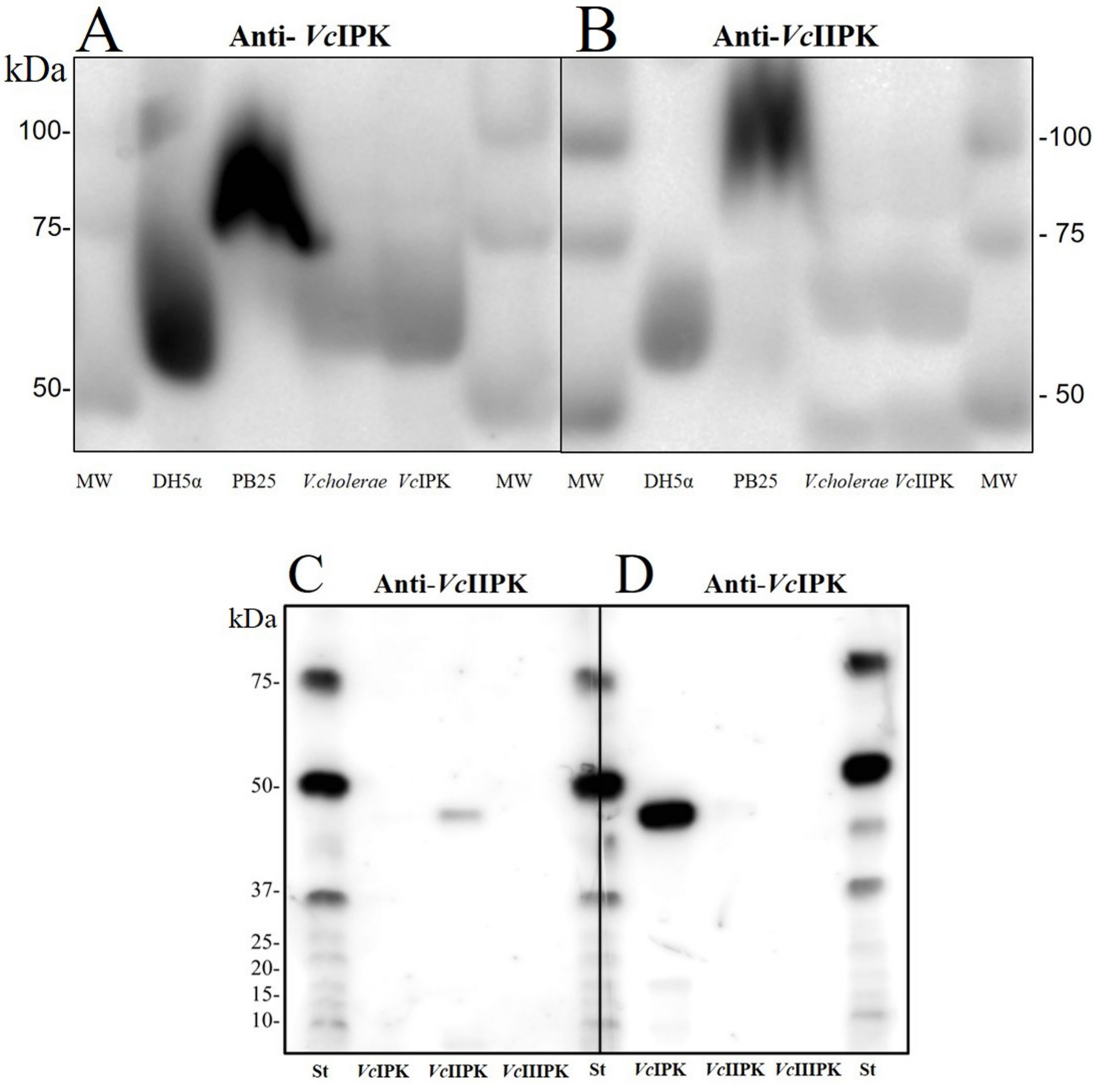
Western blot analysis of *Vc*IPK (A) and *Vc*IIPK (B) in *Vibrio cholerae* cell extracts and cross-reactivity of ployclonal anti-*Vc*IIPK (C) and anti-*Vc*IPK (D) against recombinant *Vc*IPK, *Vc*IIPK and *Vc*IIIPK. Western blot was performed with anti-*Vc*IPK (A) and anti *Vc*IIPK (B). MW are the Molecular Weight Standards (Western-C of Biorad) and lanes 2, 3 and 4 are 30 μg of cell extracts of *E*. *coli* DH5α, of *E*. *coli* PB25 and of *V*. *cholerae*, respectively. Lane 5 was loaded with purified recombinant 0.5 μg. of *Vc*IPK (A) and 1 μg of *Vc*IIPK (B). In (C) and (D) 0.5 μg of purified recombinant enzymes were loaded. *Vc*IIIPK was generously given by Dr. Gloria Hernández-Alcántara.

Western blot analyses were performed using antibodies that specifically recognize *Vc*IPK and *Vc*IIPK, as shown in Fig 8C and 8D. No cross-reactivity was found between the polyclonal antibodies and the other *V*. *cholerae* PKs. The main bands corresponded to the expected molecular mass of *Vc*IPK (53.14 kDa) (Fig 8A) and *Vc*IIPK (54.91 kDa) (Fig 8B). To show that the bands correspond to PKs, an SDS-PAGE gel including extracts of *E*. *coli* DH5α and PB25 strains was performed. *E*. *coli* DH5α strain contains the two endogenous *Pyk*F and *Pyk*A, whereas in PB25 strain, the gene of *Pyk*F has been interrupted with chloramphenicol and that of *Pyk*A with kanamycin resistance cassettes. The identification by Western blots of PK bands of *E*. *coli* extracts, *V*. *cholerae* extracts and recombinant *Vc*IPK and *Vc*IIPK recognized by anti-*Vc*IPK and anti-*Vc*IIPK display a similar migration pattern. It is relevant to mention that the recognition of *E*. *coli* PKs might be due to the high identity between these two γ-protebacteria (78% identity of *Vc*IPK vs. *Pyk*F and 69% between *Vc*IIPK and *Pyk*A). In the case of PB25 strain, the band is of a higher molecular weight due to the insertion of the antibiotic resistance cassette (chloramphenicol 25.6 in (Fig 8A) and kanamycin 29.17 in (Fig 8B)). The amount of PKs on *V*. *cholerae* extract were estimated to be 14 ± 0.2 μg/ mg of *V*. *cholerae* extract protein (*Vc*IPK) and 26 ± 0.2 μg/ mg of *V*. *cholerae* extract protein (*Vc*IIPK) by comparing the densitometry data with the lane loaded with known amounts of the corresponding recombinant PK. Lower faint bands below the main PK band may correspond to limited degradation products of the PK proteins. These data indicate that *V*. *cholerae* cells express both PKs growing in LB medium under aerobiosis.

### Determination of metabolites in *Vibrio cholerae* extracts

As described above, *Vc*IPK exhibits high activity without allosteric effector (Fru 1,6 BP), whereas *Vc*IIPK with Mg^2+^ is inactive in the absence of Rib 5-P. However, in the absence of Rib 5-P, *Vc*IIPK is capable of high activities when the divalent cation was Mn^2+^ instead of Mg^2+^. Hence, it was relevant to also consider the physiological concentrations of Fru 1,6-BP and Rib 5-P in *V*. *cholerae*. Therefore, the concentrations of these metabolites and other glycolytic intermediates were determined using cell extracts of *V*. *cholerae* (CVD 103 strain) harvested at stationary phase from LB broth (Table 6). The concentrations of Fru 1,6-BP and Rib-5P, were 0.97 mM and 4.3 mM, respectively. Other intermediates of glycolysis, Glc 6-P and Fru 6-P, were lower than those reported in cells grown at exponential phase in glucose-fed cultures [52]; whereas Rib 5-P was higher than that reported by Bennett *et al*. [52]. However the ATP determined in the same cell extracts, reached the millimolar concentrations obtained in previous reports [52] supporting the validity of the other determinations. To elucidate if both recombinant *Vc*PKs are functional under physiological metabolite concentrations found in γ-proteobacteria, experiments were performed containing 300 μM PEP [50], 550 μM ADP [52], 1.6 mM Mg^2+^_free_ [53] and either 972 μM Fru 1,6-BP for *Vc*IPK or 4.3 mM Rib 5-P for *Vc*IIPK. In these experimental conditions the activity for *Vc*IPK was 25% of *V*_max_ (122 μmol/min.mg) and the activity for *Vc*IIPK was 10% of *V*_max_ with Mg^2+^ (29 μmol/min.mg). The two forms of the enzyme appear to co-exist, at least in this *in vitro* condition. These data are coincident with a report showing that under most physiological conditions, the total pyruvate kinase activity of *E*. *coli* cell results from the contribution of both forms of the enzyme in different proportions [6, 10, 71]. On the other hand, given the concentration of Mn^2+^ in *E*. *coli* (∼10 μM) [72], it is difficult to establish the physiological role for Mn^2+^on *Vc*IIPK activity. Therefore the expression of the catalysis of *Vc*IIPK in cells at the stationary phase of growth, under aerobiosis and LB broth cell culture seems to be mainly regulated by Rib 5-P.

**Table 6:** Intracellular concentrations of metabolites in *Vibrio cholerae* CVD103 grown at stationary phase. Metabolites were determined by enzymatic coupled assays as described in Experimental Procedures. The mean and standard deviation of three independent cell extracts are shown.

Metabolite	[mM]
ATP	3.5 ± 0.4
Fru 1,6-BP	0.97 ± 0.2
Rib 5-P	4.3 ± 0.9
Glc 6-P	0.22 ± 0.02
Fru 6-P	0.02 ± 0.001

In sum the data show that *Vc*IPK and *Vc*IIPK exhibited K^+^-dependent and K^+^-independent activity, respectively. The isozymes possess different allosteric effectors, *Vc*IPK is regulated by Fru 1,6-BP (“K type” system for PEP), whereas *Vc*IIPK is activated by Rib 5-P (“mixed type” system for PEP and ADP-Mg). In contrast to the high activities expressed in *Vc*IPK in the absence of Fru 1,6-BP, *Vc*IIPK was completely dependent on Rib 5-P to exhibit high activities. However, the effect of Rib 5-P was overcome in the presence of Mn^2+^. Despite the different allosteric regulation between *Vc*IPK and *Vc*IIPK, in the presence of their respective activators both PKs exhibit similar catalytic efficiencies. Since we found that in *V*. *cholerae*, *Vc*IPK and *Vc*IIPK were co-expressed, and their allosteric effectors were present, it may be concluded that both enzymes contribute to the activity of pyruvate kinase in this bacterium. However, it cannot be discarded that the expression of PKs in *Vibrio cholerae* will change in different growing conditions and different metabolic requirements.

## Supporting information

S1 Fig Changes in *k*_*cat*_ (A) and in *k*_*cat*_/*K*_*0*.*5*_ (B) *vs* [K^+^] for PEP^3-^ of *Vc*IPK. *k*_*cat*_ and *K*_*0*.*5*_ values were obtained from curves of variable concentrations of PEP^3-^ (0.04, 0.097, 0.29, 0.87, 2.33 mM) at fixed variable concentrations of K^+^ (1.5, 3.5, 7.5, 20 and 50 mM). The assays were performed in the presence of 5 mM Fru 1,6-BP, 6.5mM ADP-Mg and 2mM Mg^2+^_free_.
(PDF)
