# Supplementary figures and images for: The contribution of two isozymes to the pyruvate kinase activity of Vibrio cholerae: One K+-dependent constitutively active and another K+-independent with essential allosteric activation
Source: PLoS One. 2017 Jul 7;12(7):e0178673. doi: 10.1371/journal.pone.0178673 (PMC5501398; doi:10.1371/journal.pone.0178673)

S1 Figure

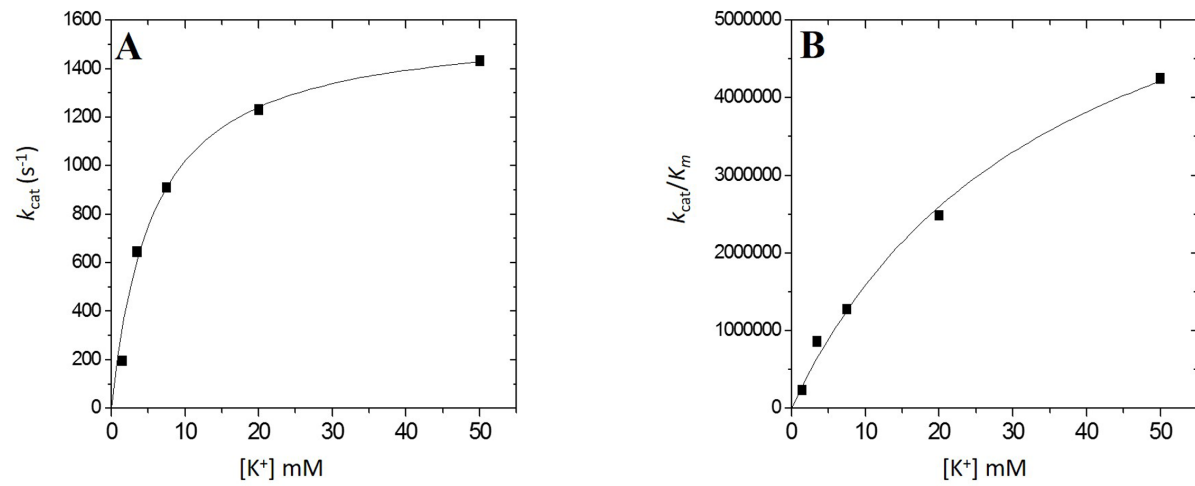

Supplement: S1 Fig — kcat and K0.5 values were obtained from curves of variable concentrations of PEP3- (0.04, 0.097, 0.29, 0.87, 2.33 mM) at fixed variable concentrations of K+ (1.5, 3.5, 7.5, 20 and 50 mM). The assays were performed in the presence of 5 mM Fru 1,6-BP, 6.5mM ADP-Mg and 2mM Mg2+free. (PDF) [file pone.0178673.s001.pdf]
